# A humanized anti-human adenovirus 55 monoclonal antibody with good neutralization ability

**DOI:** 10.3389/fimmu.2023.1132822

**Published:** 2023-03-16

**Authors:** Lei Chen, Jiansheng Lu, Junjie Yue, Rong Wang, Peng Du, Yunzhou Yu, Jiazheng Guo, Xi Wang, Yujia Jiang, Kexuan Cheng, Zhixin Yang, Tao Zheng

**Affiliations:** Academy of Military Medical Sciences (AMMS), Beijing, China

**Keywords:** human adenovirus type 55(HAdV55), monoclonal antibody(mAb), scFv-phage immune library, neutralizing antibody, conformational epitope

## Abstract

**Background:**

Human adenovirus type 55 (HAdV55) has a re-emerged as pathogen causing an acute respiratory disease presenting as a severe lower respiratory illness that can cause death. To date, there is no HAdV55 vaccine or treatment available for general use.

**Methods:**

Herein, a monoclonal antibody specific for HAdV55, mAb 9-8, was isolated from an scFv-phage display library derived from mice immunized with the purified inactived-HAdV55 virions. By using ELISA and a virus micro-neutralization assay, we evaluated the binding and neutralizing activity of mAb 9-8 following humanization. Western blotting analysis and antigen-antibody molecular docking analysis were used to identify the antigenic epitopes that the humanized monoclonal antibody 9-8-h2 recognized. After that, their thermal stability was determined.

**Results:**

MAb 9-8 showed potent neutralization activity against HAdV55. After humanization, the humanized neutralizing monoclonal antibody (9-8-h2) was identified to neutralize HAdV55 infection with an IC50 of 0.6050 nM. The mAb 9-8-h2 recognized HAdV55 and HAdV7 virus particles, but not HAdV4 particles. Although mAb 9-8-h2 could recognize HAdV7, it could not neutralize HAdV7. Furthermore, mAb 9-8-h2 recognized a conformational neutralization epitope of the fiber protein and the crucial amino acid residues (Arg 288, Asp 157, and Asn 200) were identified. MAb 9-8-h2 also showed favorable general physicochemical properties, including good thermostability and pH stability.

**Conclusions:**

Overall, mAb 9-8-h2 might be a promising molecule for the prevention and treatment of HAdV55.

## 1 Introduction

The common acute respiratory disease (ARD) pathogen, human adenovirus (HAdV), is an envelope-free, icosahedral, double-stranded DNA virus in the Mastadenovirus genus (family Adenoviridae) (1–3). There are over 111 genotypes of HAdV, forming seven species (HAdV-A to G), which have been identified and defined using genomics (http://hadvwg.gmu.edu/) (4). A few HAdVs, including HAdV3, HAdV4, HAdV7, and HAdV55, cause serious infections, particularly in specific populations, including children, the elderly, and persons with severely weakened immune systems, although most HAdVs only cause mild or restricted infection (5). Among them, HAdV55 is one of the most important serotypes causing severe acute respiratory infections in many countries worldwide (6). In numerous provinces of China, re-emerging HAdV55 has been linked to severe community-acquired pneumonia in civilian and military populations (1, 7–10). The clinical signs and symptoms of HAdV55 infection include pneumonia, bronchitis, sore throat, myalgia, cough, and high fever, and is a potentially lethal condition. There is no herd immunity and HAdV55 has a higher propensity to produce severe ARD than other adenoviruses; therefore, HAdV55 could spread widely and create catastrophic epidemics (11). Moreover, there is no effective antiviral therapy to prevent or treat adenovirus infection. Therefore, the development of drugs to treat HAdV55 infection had become urgent.

Passive immunotherapy, using the effector activities of antibodies, can promote the direct clearance of virus-producing cells, viral antigens, or circulatory viruses (12, 13). Antibodies that neutralize viruses showed promise as preventative or therapeutic measures against viral infections (14). Ebolavirus and respiratory syncytial virus infections could both be treated with intravenously administered antibodies (15, 16). The body could develop long-lasting immunity to the same type of virus after contracting an adenovirus by the creation of neutralizing antibodies (17). Therefore, a major advance in the prevention and management of viral infection is the use of adenovirus neutralizing antibodies for passive immunotherapy.

Hexon, penton base, and fiber were the three main proteins that make up the HAdV capsid. The most prevalent capsid protein, the hexon protein, attract cytoplasmic dynein, which is essential for the movement of viral capsids along microtubules (18–20). The fiber protein protrudes from each of the twelve vertices of the icosahedral capsid and mediates selective and high-affinity binding to major cellular receptors (17). The twelve vertices of the capsid are formed by the penton base, which promotes clathrin-mediated endocytosis (21, 22). Any of these important capsid proteins could be used to produce anti-adenovirus antibodies. The variations in the frequency and makeup of these HAdV-specific neutralizing antibodies are unknown. Serotype-specific neutralizing antibodies for HAdV3, 5, 7, 14, or 55 primarily targeted the hexon protein (23). Anti-hexon neutralizing antibodies could internalize the virus without accompanying viral gene expression. Anti-hexon antibodies neutralize HAdV by cross-linking to the hexon envelope, thereby preventing viral decapsidation and viral DNA from entering the nucleus (24). The fiber protein could stimulate the production of antibodies that were cross-neutralizing to HAdV14 and HAdV55. It was demonstrated that both polyclonal and monoclonal anti-fiber neutralizing antibodies could exert antiviral effects by cross-linking fibers on individual viral particles or by inhibiting the binding of AdV5 fiber knobs to their cell attachment receptors (25, 26). Anti-penton antibodies significantly aided in the neutralization of HAdV infection, primarily at the stage of virus internalization (22).

The present study comprised the construction of a single-chain variable fragment (scFv)-phage display library from mice immunized using purified HAdV55, from which we identified 14 clones specific for HAdV55 with different sequences. Then, we successfully identified five mAbs with potent neutralizing activities. MAb 9-8, which had the highest affinity and neutralizing activity among these five candidate antibodies, was humanized and the humanized mAb 9-8-h2 retained biological activity similar to that of the parental antibody. The humanized mAb 9-8-h2 significantly inhibited the cytopathic effect of HAdV55 virus *in vitro* at low concentrations and displayed good stability. Thus, mAb 9-8-h2 showed promise as an effective prophylactic treatment and therapy for HAdV55 infection.

## 2 Materials and methods

### 2.1 Viruses and cell lines

Hiam A549 cells were grown in Dulbecco’s modified Eagle medium (DMEM; Life Technologies, Carlsbad, CA, USA) with 10% fetal bovine serum (FBS; Gibco, Grand Island, NY, USA). FreeStyle^TM^ 293 Expression Medium (Gibco) was used to culture FreeStyle^TM^ HEK 293-F cells (Invitrogen, Waltham, MA, USA). Cells were grown in a 5% CO_2_ atmosphere at 37°C. HAdV55 (GenBank accession no. AF532578.1), HAdV4 (GenBank accession no. AF532578. 1) and HAdV7 GZ6965 strain (GenBank accession no. AF532578.1) were maintained in our laboratory (27). All HAdVs were grown in A549 cells cultured in DMEM with 2% FBS. When 80–90% of the cells showed a cytopathological effect (CPE) characteristic of HAdV infection, the viral cultures were harvested and freeze-thawed three times. The viral supernatants were collected by centrifugation for purification by calcium chloride gradient centrifugation (28). The 50% tissue culture infective dose (TCID50) was calculated to determine adenovirus titers according to the classical Reed-Muench method (29).

### 2.2 scFv-phage antibody library construction

A previously published method (27) was used to produce and screen scFv colonies directed against HAdV55. Briefly, we immunized female BALB/c mice (6-8 weeks old) intraperitoneally using purified HAdV55 (in phosphate buffered saline (PBS)) mixed with a 1/10 volume of aluminum adjuvant every 14 days for a total of 4 times. Three days after each mouse was intravenously injected with 25 μg of HAdV55, their blood was collected and serum was isolated to detect the antibody titers in mouse serum. Pre-immune mouse serum samples were collected for use as negative controls. Mice were killed and their spleens were harvested one week after the final booster immunization. Isolated spleen cells were subjected to total RNA extraction, which was reverse transcribed to cDNA using the Invitrogen Superscript III First-strand synthesis system for RT-PCR reverse transcription kit (K1621, Thermo Scientific, USA). The cDNA was used as a template to amplify the antibody heavy chain variable region (VH) and light chain variable region (VL). Primer sets for amplification of the VH and VL regions were designed with reference to the literature (30). The scFv gene fragment was then obtained using overlay-extended PCR amplification, digested using Nco I/Not I, and ligated into the NEN-SCFV phage display vector. The ligation mixture was transformed into competent *E. coli* TG1 cells using electroporation. To determine the library size, transformed cells were titrated on 2-YT agar plates, and we carried out colony PCR on selected colonies to verify the presence of DNA inserts in the vector. Harvested cells containing the verified scFv antibody gene library were harvested pooled, aliquoted, and stored at −80°C.

### 2.3 Selection of the scFv-phage antibody library

The HAdV55-specific phage antibody library was screened using a solid phase screening strategy using purified HAdV55 as the antigen. Purified HAdV55 in PBS was coated onto an immunotube at 4°C overnight and blocked using 3% bovine serum albumin (BSA) in PBS (50 mM Tris-HCl pH 7.5, 150 mM NaCl) overnight at 4°C. An aliquot of the phage library (5.0×10^11^ plaque-forming units (pfu) per mL) was added and the tube was incubated for another 2 h at 37°C. Tubes were washed using PBST (PBS containing 0.1% Tween-20), and 1 mL of 0.1 M glycine-HCl (pH 2.2) was used to elute the bound phage, which was neutralized using 50 μL 1 M Tris-HCl (pH 9.0). Eluted phage was infected into *E. coli* TG1 cells for amplification, followed by two more rounds of the panning process. The final eluted phages were identified using PHAGE-enzyme linked immunosorbent assay (ELISA). Positively selected phages were amplified and the nucleotide sequence of the resulting scFv was determined. The identification of the sequences of genes encoding VH and VL were performed using the IMGT database (HTTP://www.imgt.org/IMGTlect/).

### 2.4 Cloning, expression, and purification of the antibody

The full-length VL and VH chain genes of the positively selected phages and humanized antibodies were amplified by PCR. Restriction endonucleases *Sal I/Nhe I* or *Sal I/Nar I* were used to digest the PCR amplicons and the resultant fragments were ligated separately into vectors pTSEG1n or pTSEK, each comprising a human immunoglobulin constant gene. The constructed expression plasmids were cotransfected into FreeStyle^TM^ HEK293-F cells using the transfection reagent FectoPRO DNA Transfection Reagent (Polyplus, Illkirch, France; 116-001). After 4 days, a HiTrap protein G apparatus (28-4082-60, (GE Healthcare Bio-Sciences AB, Uppsala, Sweden)) was used to harvest and purify the antibodies from the antibody-containing supernatants, following to the supplier’s guidelines. SDS-PAGE was employed to determine the purity of the antibodies, and a NanoDrop spectrophotometer (Thermo Fisher Scientific, Waltham, MA, USA) was used to evaluate the antibody concentration.

### 2.5 ELISA

A microtiter 96-well plate (9018, Costar, Washington, DC, USA) was coated with 200 ng/well of purified virus and incubated at 4°C overnight. The plate was added with 200 µL/well of 2% (w/v) Non-Fat Dry Milk (NFDM, Bio-Rad, Hercules, CA, USA) in PBS at 37°C for 2 h and then incubated for 1.5 h at 37°C with 100 µL/well of antibodies at various dilutions in Milk-PBS. Plates were washed three times with PBST and then incubated with goat anti-mouse/human horseradish peroxidase (HRP)-conjugated IgG antibody (1: 4,000, v/v) at 37°C for 45 min. Finally, the plates were washed six times using PBST, and the color was developed using o-Phenylenediamine (OPD) chromogen substrate (Sigma, UK). The reaction was stopped using 50 µL of 2 M H_2_SO_4_ and the OD492/OD630 determined in a microtiter plate reader (iMark, Bio-Rad).

### 2.6 Virus micro-neutralization assay

A549 cells (100 µL containing 2×10^5^ cells/mL) were seeded in the wells of 96-well plates and incubated at 37°C overnight in a 5% CO_2_ atmosphere. Purified mAbs were diluted serially by 2-fold using DMEM, and 50 µL samples of each dilution were added with 50 µL HAdVs at 100 TCID50. The virus-antibody mixtures were incubated for 1 h at 37°C. The mixtures were then transferred into 96-well plates comprising A549 cell monolayers at 85–95% confluence and cultured in DMEM containing 2% FBS at 37°C for 1 h. A microscope was used to observe the infected cells and then the viability of the A549 cells was determined using a Cell Counting Kit-8 (CCK-8) assay (Vazyme Biotech, Nanjing, China) according to the manufacturers protocol. After washing the 96-well cell culture plate twice with sterile PBS, DMEM medium (containing 10% FBS and 10% CCK-8 reagent) was added at 100 μL/well and incubated for 2.5 h at 37°C in a cell incubator. The absorbance value at 450 nm was then measured using a microtiter plate reader. The neutralizing titers and concentrations of the mAbs were used to calculate the half-maximal inhibitory concentrations (IC_50_).

### 2.7 Western blotting analysis

Briefly, purified antigens were separated using SDS-PAGE (together with prestained protein markers (NEB, Hitchin, UK)) and then electroblotted onto a polyvinylidene fluoride (PVDF) membrane for immunoblotting. The membrane containing the transferred proteins was blocked using 5% (w/v) NFDM in TBS buffer for at least 2 h. After blocking, a mAb or antiserum was added and incubated overnight in a refrigerator at 4°C. The membrane was then incubated with goat anti-human/mouse horseradish HRP-conjugated IgG antibody (1:4,000, v/v) (ZSGB-BIO Beijing, China). Western HPR Substrate Peroxide solution (Millipore, Billerica, MA, USA) was used to detect the signals.

### 2.8 Antigen-antibody molecular docking analysis

The antibody and antigen sequence (fasta format) were submitted to AlphaFold2 (31), which was operated in the multimer mode and monomer mode. The top ranked structure predicted using local distance difference test (pLDDT) score model was chosen as best structure. The antibody PDB residues were renumbered using the Chothia antibody-numbering scheme (32). The renumbered PDB residues were then submitted to the Antibody_H3 module in Rosetta 3 (33) with default settings to optimize the complementarity determining region 3 (CDR3) conformation. The top 1000 conformations were generated and ranked as Antibody_H3 scores. HADDOCK (34) was employed to simulate antibody-antigen docking. HADDOCK performed rigid body docking to generate 10000 conformations. The top 1000 conformations with minimum energy score were selected to perform flexible refinement and energy minimization refinement. According to the Root Mean Square Deviation (RMSD), the conformations were divided into different clusters. The top five structures from the Antibody_H3 results with antigen structures were submitted to HADDOCK to perform five times docking. The best conformation of the antibody-antigen complex was selected from the largest conformation cluster by visual inspection.

### 2.9 Thermostability assessment

The candidate mAb’s thermostability was assessed utilizing a UNcle (UNchained Laboratories) detection approach based on fluorescence, dynamic light scattering (DLS), and static light scattering (SLS). A mAb sample was diluted in PBS to 3333 nM in several formulations and tested in duplicate. Between 25 and 95°C, a heating rate of 0.25°C/minute was observed. The best analytical technique was determined to be the barycentric mean (BCM) of the fluorescence intensity. The SLS data were simultaneously gathered while the SLS assays were measured at 266 nm. The greatest point of the first derivative of the melting curve was defined as the melting temperature (Tm). The first positive data point above the baseline of the first derivative of SLS was used to define aggregate temperatures (Tagg). Finally, Tm and Tagg from the DLS assay were analyzed and calculated using UNCLE Analysis Software (Unchained Labs, Pleasanton, CA, USA) (35).

### 2.10 Statistical analysis

The mean and standard deviation for all quantitative data are shown. One-way ANOVA or a t-test were used to determine the statistical significance of the differences between groups. P-values under 0.05 were used to determine the statistical significance of the differences. GraphPad Prism 5 was used to analyze all of the data (GraphPad Inc., La Jolla, CA, USA).

## 3 Results

### 3.1 Panning and screening of anti-HAdV55 scFv clones

**Figure 1:**
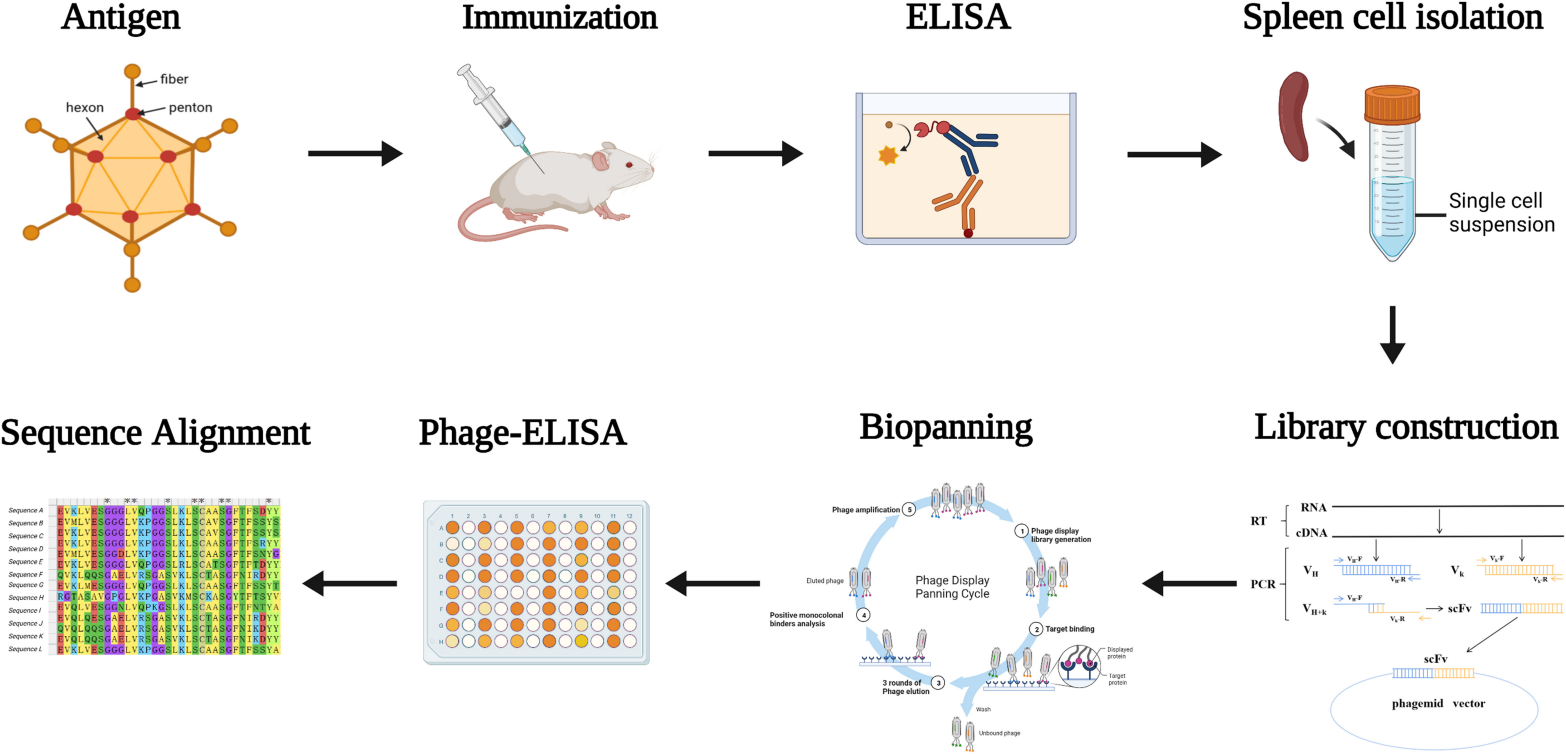
Diagram of the strategy for immunization and screening used to isolate mAbs recognizing HAdV55. Mice were immunized with inactivated and purified HAdV55 virus, and serum was collected to determine antibody titer. Following that, RNA from mouse spleen was isolated and reverse-transcribed into cDNA. The light and heavy chain variable region genes (VL and VH) of the antibodies were amplified using cDNA as a template. The gene fragment encoding the single-chain fragment variable (scFv) antibody was obtained by amplifying VL and VH using overlapping extension PCR, and scFv was then cloned into the phage vector to create the scFv phage display library. After that, the scFv phage display library was screened three times with purified HAdV55 as the target. Using Phage-ELISA, positive phage clones were identified. The screened positive phage clones were subjected to a sequence analysis.

To obtain neutralizing antibodies with high specificity, diversity and affinity, mice were immunized using purified inactivated-HAdV55 (**Figure S1**). The immune sera showed effective and specific serological activity against HAdV55 binding, at a titer of 409600, compared with that of the pre-immune serum (**Figure S2**). We then constructed a phage display library, which had > 2×10^8^ colony-forming units (CFU). After two rounds of phage display biopanning, the binding partners of HAdV55 were selected using PHAGE-ELISA, which identified 130 out of 234 clones as positive clones (**Figure S3**). Sequencing of the 130 clones allowed us to discard duplicate sequences based on their amino acid sequence alignment. Notably, 14 HAdV55-specific clones with different sequences were identified. The process is shown in **Figure 1**.

### 3.2 Production and characterization of chimeric antibodies

**Figure 2:**
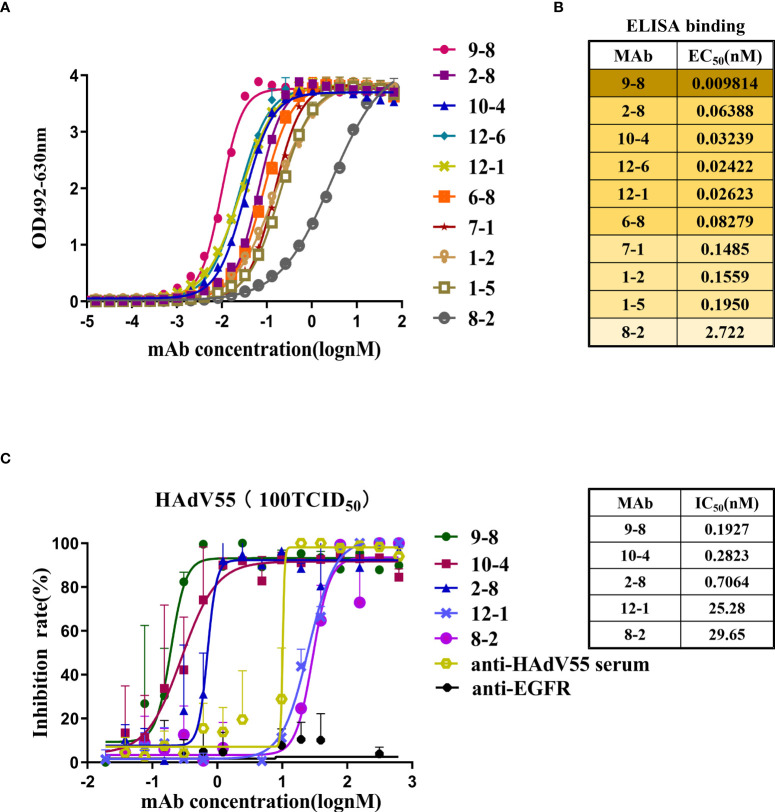
Profiles of the binding and neutralization of mAbs toward HAdV55. **(A)** Curves showing the binding between all mAbs and HAdV55. **(B)** ELISA data-generated heatmap showing the binding between the mAbs and HAdV55 (n = 3 independent experiments). **(C)** Neutralization assay of the chimeric antibodies, 9-8, 10-4, 2-8, 12-1, and 8-2 toward viruses. Data are shown as the mean ± SD (n = 3 independent experiments). Nonlinear regression analysis was used to obtain the IC_50_ value.

The sequences encoding the heavy chain (VH) and light chain (VL) of selected scFv clones were amplified and ligated into the antibody expression vectors pTSE-G1n and pTSE-K, respectively, to express human-mouse chimeric IgG1. Ten mAbs were successfully expressed and purified using affinity chromatography. Next, we investigated the properties of these antibodies, including their binding affinity and neutralizing activity. HAdV55-bound antibodies were screened using ELISA (**Figure 2A**). The EC_50_ values ranged from 0.0098 to 2.722 nM, indicating strong binding to HAdV55 by all the mAbs (**Figure 2B**). Next, the neutralizing abilities of the mAbs toward HAdV55 were determined. The results showed that 5 out of 10 candidate antibodies showed significant blocking effect on HAdV55 infection of A549 cells (**Table S1**). The inhibition rates of the five candidate mAbs (9-8, 10-4, 2-8, 12-1 and 8-2) were calculated using normalization against a control sample (**Figure 2C**). The five mAbs displayed dose-dependent neutralizing protection, with IC_50_ values below 29.65 nM, including a minimum IC_50_ of 0.1927 nM. Through a comprehensive evaluation of various parameters, we finally selected mAb 9-8, which had the best binding activity and neutralization activity, as a candidate antibody for subsequent studies.

### 3.3 Production, binding, and neutralization of humanized antibodies

**Figure 3:**
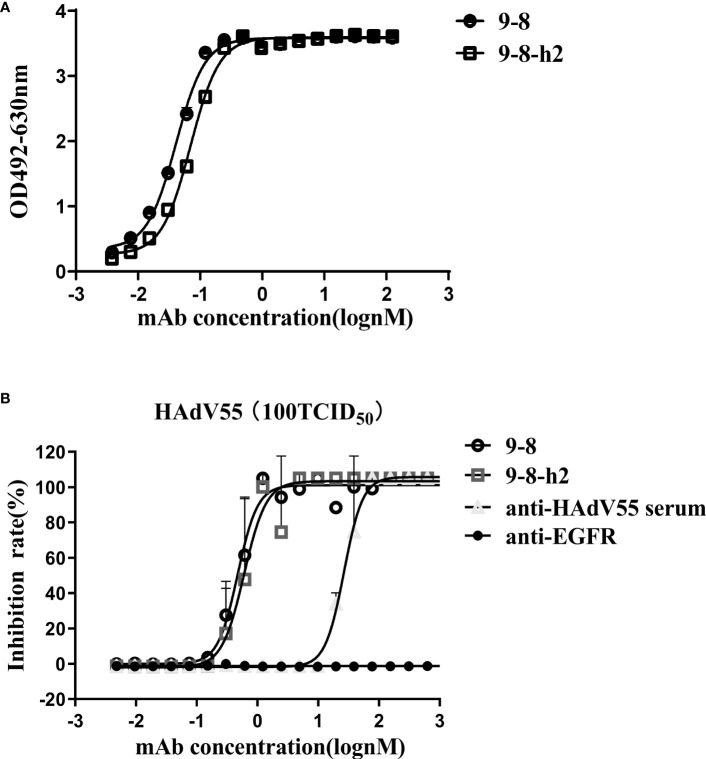
Humanized mAb 9-8-h2 binding and neutralization abilities. **(A)** Humanized mAb 9-8-h2 binding to HAdV55, as assessed using ELISA. **(B)** Neutralization assay of humanized mAb 9-8-h2. Data are shown as the mean ± SD (n = 3 independent experiments). Nonlinear regression analysis was used to obtain the IC_50_ value.

Hu-mAb, an antibody humanization tool, reduced the likelihood of immunogenicity in murine antibody sequences (36). The mouse-derived antibody 9-8 was humanized by Hu-mAb (http://opig.stats.ox.ac.uk/webapps/newsabdab/sabpred/humab). In order to humanize antibody variable region sequences, mutations that improve the “human” score were created, but only in the framework region, leaving the CDR residues unaltered to maintain antibody binding characteristics (**Table S2**). The humanized VH and VL sequences were fused those of the human VH and LH constant domains encoded in pTSE-G1n and pTSE-K vectors, respectively, to construct the humanized mAb 9-8-h2. SDS-PAGE demonstrated that the protein purity of mAb 9-8-h2 was higher than 95% (**Figure S4**). To compare the binding activity between the humanized mAb 9-8-h2 and the chimeric antibody mAb 9-8, the EC_50_ for antibody binding to HAdV55 was calculated according to the ELISA results. As shown in **Figure 3A**, mAb 9-8-h2 bound to HAdV55 with an EC_50_ of 0.0701 nM, which was comparable to that of mAb 9-8 (0.0410 nM). Furthermore, the neutralizing activity of the antibody was assessed using a micro-neutralization assay. The humanized mAb 9-8-h2 demonstrated a neutralization ability that was slightly lower than that of the human-mouse chimeric mAb 9-8; however, the humanized mAb 9-8-h2 could still neutralize HAdV55 infection with an IC_50_ of 0.6050 nM (**Figure 3B**).

### 3.4 Cross-reactivity of the humanized mAb 9-8-h2 against other types of HAdVs

**Figure 4:**
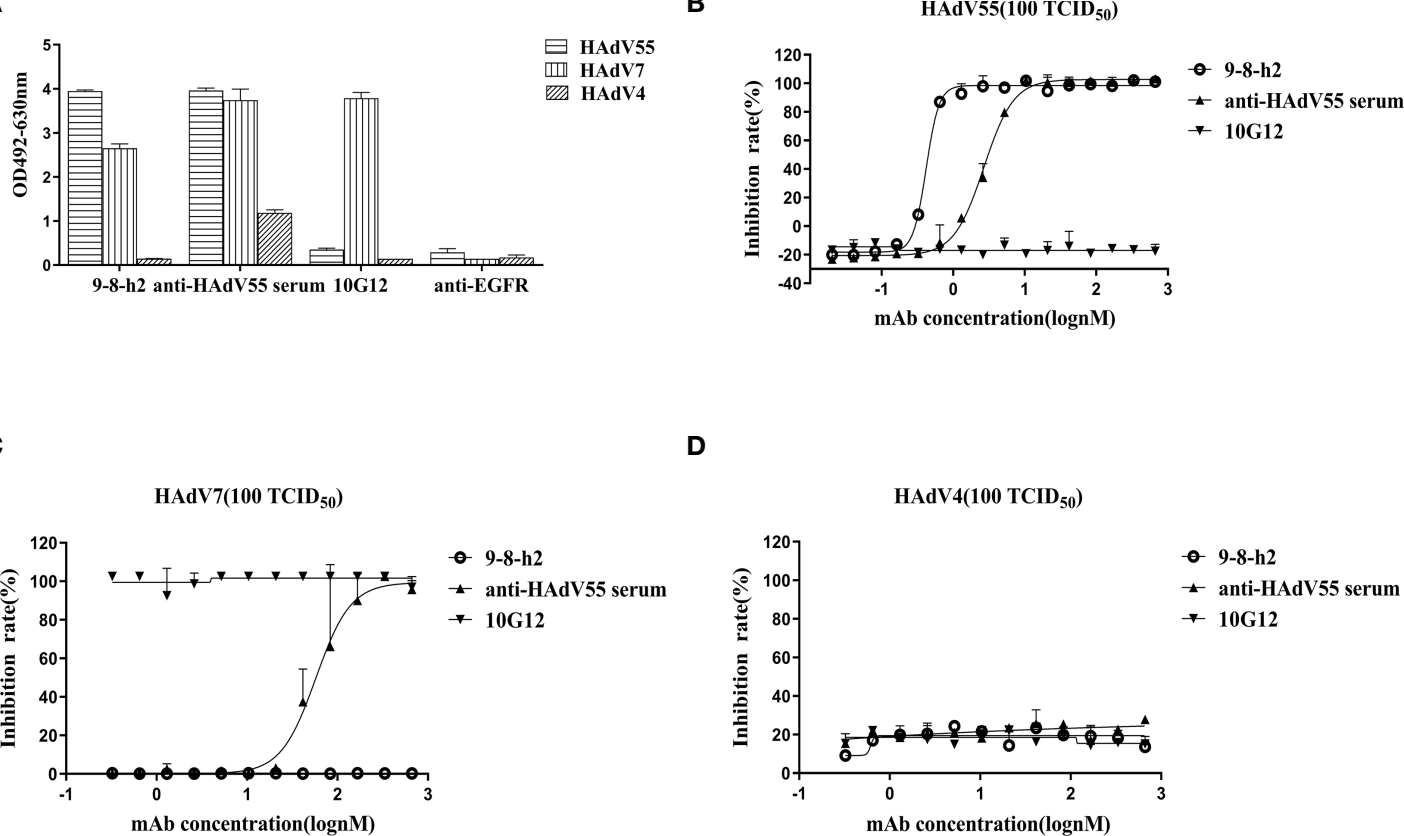
Humanized mAb 9-8-h2 cross-reacts with several types of HAdV. ELISA detection of humanized mAb 9-8-h2 reacting with purified virions from HAdV4, HAdV7, and HAdV55 **(A)**. n = 3 independent experiments; data are shown as the means ± SD. Humanized mAb 9-8-h2 was assessed for NAb titers to HAdV55 **(B)**, HAdV7 **(C)** or HAdV4 **(D)** viruses using an *in vitro* micro-neutralization test.

To identify the cross-reactivity of antibody mAb 9-8-h2, purified HAdV55, HAdV7, and HAdV4 virions were employed as coating antigens for ELISA. Two unrelated antibodies (anti-EGFR antibody (anti-EGFR) and anti-HAdV7 antibody (10G12)) served as the negative controls and the anti-HAdV5 serum served as the positive control. As shown in **Figure 4A**, mAb 9-8-h2 interacted with HAdV55 and HAdV7, but not with HAdV4. In comparison to mAb 9-8-h2 to HAdV55, mAb 9-8-h2’s binding OD value to HAdV7 was lower. To examine mAb 9-8-h2’s potential for cross-neutralization, we performed adenovirus neutralization experiments *in vitro*. A549 cells were infected with HAdV7, HAdV4, and HAdV55 with 100 TCID50. The humanized mAb 9-8-h2 could neutralize HAdV55, but not HAdV7 or HAdV4 (**Figures 4B–D**). According to the results of ELISA, mAb 9-8-h2 bound to HAdV7, but could not neutralize HAdV7, which indicated that the epitope of mAb 9-8-h2 recognized on HAdV7 might not be neutralizing.

### 3.5 Humanized mAb 9-8-h2 binds the fiber protein

**Figure 5:**
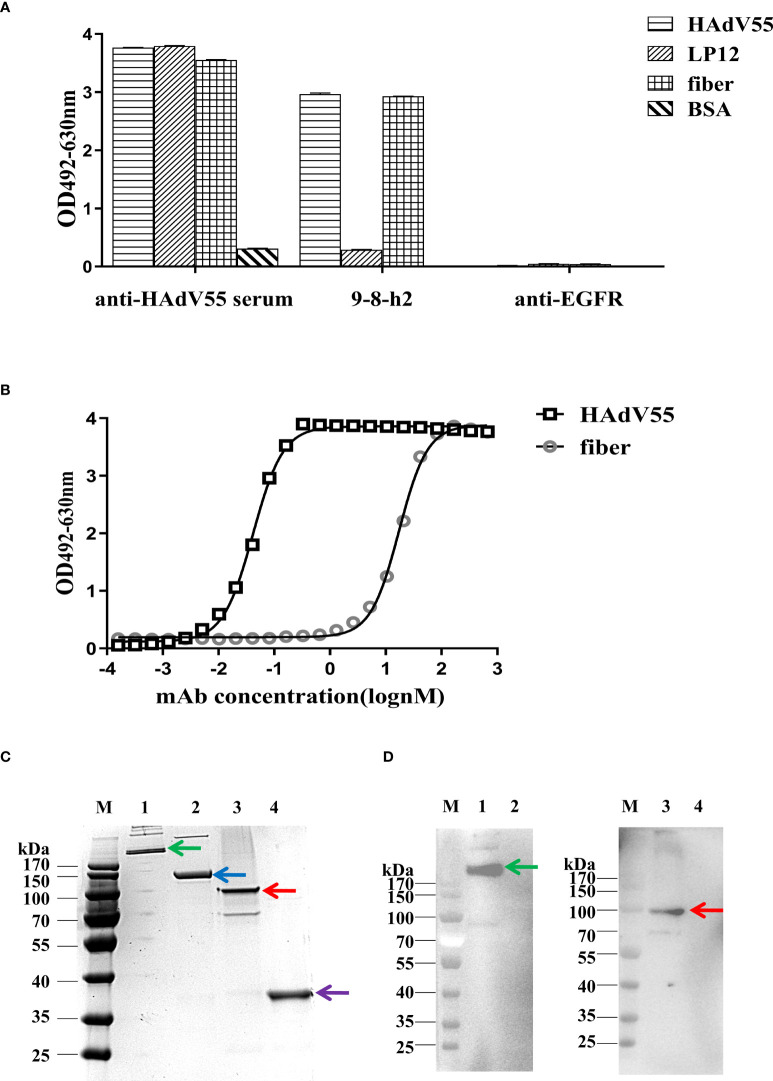
Binding of humanized mAb 9-8-h2 to the fiber of HAdV55. **(A)** Binding of various antigens by mAb, assessed using ELISA analysis (n =3 independent experiments). Data are shown as the means ± SD. **(B)** ELISA-derived affinity curve for binding between serially diluted (starting concentration of 66.7 nM) humanized mAb 9-8-h2 and the fiber (200 ng/well). **(C)** SDS-PAGE analysis of the purified HAdV55 and recombinant HAdV55 fiber. Purified HAdV55 virions and recombinant fiber proteins were heated at 98°C (“Reducing”) or stored at room temperature (“Non-reducing”) for 5 min with loading buffer. **(D)** The polymeric form of the HAdV55 fiber is recognized by the mAbs, as assessed using western blotting. A 300 ng/well sample of purified HAdV55 virions and recombinant fiber protein were loaded/used in SDS-PAGE and WB analysis. Primary antibody (6667 nM) was diluted 1:1,000. M, prestained protein markers; 1, Purified HAdV55 virions (Non-reducing, Green arrow); 2, Purified HAdV55 virions (Reducing, Blue arrow); 3, recombinant fiber protein (Non-reducing, Red arrow); 4, recombinant fiber protein (Reducing, Purple arrow).

Hexon is a crucial protective antigen for the generation of neutralizing antibodies, and the fiber protein contains certain neutralizing epitopes, according to earlier research (37). ELISA and immunoblotting analysis were then carried out to discover the epitope recognized by mAb 9-8-h2. The hexon LP12 fragment and fiber-encoding genes were amplified by PCR and introduced into the pTIG-TRX vector, respectively. *Escherichia coli* BL21 (DE3) cells were transformed with the pTIG-TRX-LP12/fiber plasmid to produce the His-tagged fusion protein, which was described in our previous study (27). As coating antigens for ELISA, HAdV55 virions, fragment LP12 (comprising loop1 and loop2 of the hexon), and fiber were employed. The results demonstrated that HAdV55 virions and fiber, but not LP12, were bound by mAb 9-8-h2 (**Figure 5A**). After coating the ELISA assay plates with fiber, the affinity of mAb 9-8-h2 for the fiber protein was calculated. The results revealed that mAb 9-8-h2 had a lower affinity for fiber than for HAdV55, with a binding activity of 17.21 nM (**Figure 5B**). Additionally, we performed immunoblot analysis under reducing and non-reducing conditions, with or without beta-mercaptoethanol (β-ME). The HAdV55 fiber protein retained its trimeric form in SDS-PAGE loading buffer with 10% SDS but without β-ME and heating. Theoretically, the HAdV55 fiber monomers had a molecular weight of about 35 kDa, while the theoretical molecular weight of the trimer was about 105 kDa (**Figure 5C**). The humanized mAb 9-8-h2 recognized HAdV55 (lane 1) and trimeric HAdV55 fiber (lane 3) under non-reducing conditions, but did not recognize HAdV55 (lane 2) and monomeric HAdV55 fiber (lane 4) under reducing conditions (β-ME plus heating at 98°C) (**Figure 5D**). These results suggested that the conformation of the epitopes on the HAdV55 fiber homotrimer affected their recognition by mAb 9-8-h2.

### 3.6 Antibody structures and computational docking studies

**Figure 6:**
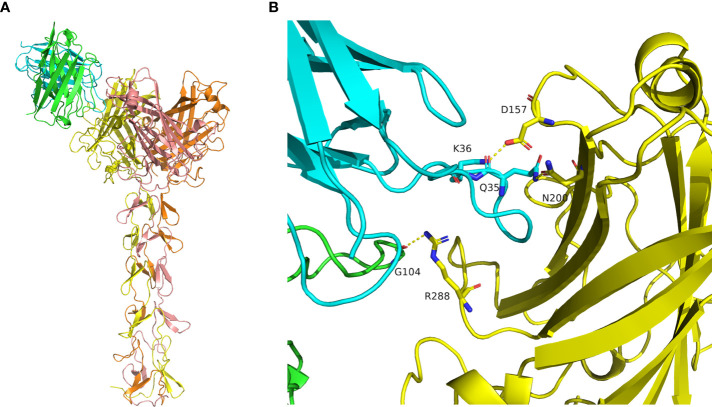
Molecular docking of mAb 9-8-h2 to the Fiber antigen. **(A)** Predicted structure of the antibody-antigen complex by HADDOCK3. The heavy chain is marked in green, and the light chain is marked in cyan. The antigen homotrimer is marked in yellow, orange, and salmon. **(B)** Binding mode of the antibody-antigen interaction surface.

The structure of mAb 9-8-h2 was predicted using the AlphaFold2 program based on its protein sequence. The best predicted model was reasonable according to its pLDDT score of 0.921. The conformation of CDR3 was stretched out in the predicted model, which is rare in antibody structures (**Figure S5A**). The antibody conformation was further optimized using the Antibody_H3 module of Rosetta 3. The top five of 1000 conformations generated by Antibody_H3 were selected according to their ranking scores (**Figure S5B**). The heavy chain CDR3 structure of those five antibody conformations were reasonable in antibody binding, in which they adopted a bending pose. Those five hits were used for antibody-antigen complex prediction (**Figure S6**; **Table S4**). The structure of the fiber homotrimer was predicted using AlphaFold2 based on its protein sequence (**Figure S5C**). The best predicted model was reasonable according to its pLDDT score of 0.857 and was used for antibody-antigen complex prediction. The structure of the antibody-antigen complex was predicted using HADDOCK3. According to the predicted conformation clustering analysis, antibody_0307 bound with the receptor binding domain of the antigen was the best predicted model, which belonged to the largest cluster containing eight similar conformations (**Figures 6A**, **S7**; **Table S5**). Gly 104 of heavy chain CDR3 bound with antigen Arg 288 *via* a hydrogen bond. Besides, the Lys 36 of CDR1 in the light chain formed a salt bridge with Asp157 of the antigen, and Gln 35 of CDR1 in the light chain formed a hydrogen bond with Asn 200 of the antigen (**Figure 6B**).

To corroborate the computational docking results of HAdV55 and mAb 9-8-h2 was correct, the binding ability of mAb 9-8-h2 to recombinant HAdV55 fiber proteins which were mutated at Arg 288, Asp 157, and/or Asn 200 by alanine mutagenesis. Arg 288, Asp 157, and/or Asn 200 was mutated to alanine respectively. Fiber mutants that altered the key amino acids were constructed and purification (**Figure S8A**). In ELISA, the mAb 9-8-h2 recognized fiber and three fiber single site mutants (D157A, N200A or R288A), but did not recognize fiber-mut (D157A, N200A and R288A). Analysis of the binding affinity of mAb 9-8-h2 to fiber revealed an EC_50_ value of 11.49 nM, which was range from 2.95 to 8.79 fold higher than that of the three fiber single site mutants (**Figure S8B**). These results suggested that Arg 288, Asn 200, especially Asp 157 were critical to fiber/mAb 9-8-h2 binding.

### 3.7 Biophysical and stability/forced degradation study of mAb 9-8-h2

**Figure 7:**
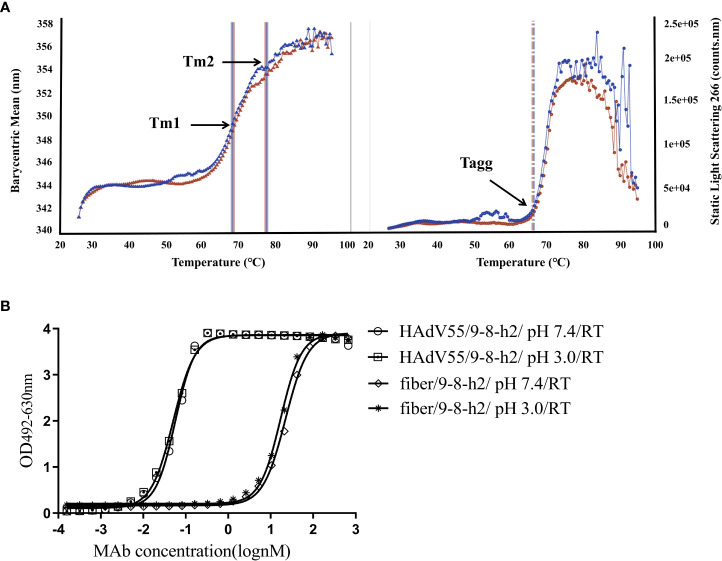
Physical properties of humanized mAb 9-8-h2. **(A)**Tm and Tagg with optional DLS detection of the developed characteristics of the thermostability properties of mAb 9-8-h2. The data are from two independent replicate experiments. **(B)** ELISA of mAb 9-8-h2 and HAdV55/fiber binding. MAb 9-8-h2 was incubated under different pH value (pH3 or 7.4).

Pharmaceutical quality control requires the characterization of the biophysical characteristics of antibodies. The creation of new therapeutics largely depends on the aggregation behavior of biological molecules. Thermostability was assessed and the graph demonstrated that the melting temperature (Tagg) for mAb 9-8-h2 was 65.70°C, with a melting temperature for the first transition (Tm1) of 67.47°C, and a melting temperature for the second transition (Tm2) of 76.70°C (**Figure 7A**; **Table S3**). Under normal conditions (pH 7.4, RT), and under pH stress (pH 3, RT), mAb 9-8-h2 exhibited no significant aggregation (**Figure 7B**). The humanized mAb 9-8-h2 had good thermostability and aggregation resistance, indicating that it possessed favorable general physicochemical properties necessary for its development as a passive immune agent.

## 4 Discussion

To date, only one human mAb, 3-3E, has been isolated from a single plasma cell contained in HAdV-7 infected patient-derived peripheral blood mononuclear cells using the single B-cell PCR technique (23). Most of anti-HAdV neutralizing antibodies have been developed from mice (38). Tian et al. obtained an mAb with broad neutralizing activity against HAdV types 55, 14p, 7, and 11 using the hybridoma fusion technique with recombinant type 11 fiber knob (39). In our preliminary study, we generated 10G12, a novel anti-HAdV7 antibody, by immunizing a mouse to construct an scFv-phage immune library. We found that 10G12 could specifically bind the hexon protein of HAdV7 and had effective neutralization activity against HAdV7 (27). Mouse-derived monoclonal antibodies could produce human anti-murine antibody (HAMA) reactions because of their immunogenicity, which might be life-threatening to the patient; therefore, they require humanization before therapeutic use. However, the humanization of anti-HAdV murine antibodies has not yet been reported. Herein, an scFv-phage immune library was obtained from mice immunized using HAdV55, in which a potent humanized neutralizing antibody, mAb 9-8-h2, was identified that could inhibit HAdV55 infection *in vitro*. To the best of our knowledge, this is the first report of a humanized mAb that can neutralize HAdV55.

It is generally accepted that HAdV mAbs are serotype-specific, and some earlier investigations demonstrated that they exhibited little or no cross-reactivity with other species of HAdV. Meanwhile, few studies have examined the cross-neutralization of various strains of the same HAdV species (39). Considering that there are more than 111 HAdV genotypes, we selected HAdV7, which belongs to the same species as HAdV55 but is a different serotype, and HAdV4, which is a different species, to detect the cross-reactivity of mAb 9-8-h2. The ELISA results showed that mAb 9-8-h2 interacted with HAdV55 and faintly with HAdV7, but not with HAdV4 (**Figure 6A**). Importantly, *in vitro* neutralization experiments showed that mAb 9-8-h2 neutralized HAdV55, but not HAdV7 and HAdV4 (**Figures 6B–D**). Rong et al. suggested that antibody 10G12 cannot neutralize HAdV55, and showed little cross-reactivity, likely *via* interactions with non-neutralizing epitopes (27). The current study showed that the sequences of mAb 9-8-h2 that recognize the antigenic epitopes of HAdV7 and HAdV55 had some homology; however, the differences in certain key amino acids resulted in mAb 9-8-h2 being able to neutralize HAdV55 but not HAdV7.

When humans were immunized with recombinant adenovirus capsid proteins, anti-fiber antibodies appeared first, followed by anti-penton antibodies, and then anti-hexon antibodies (40). Studies have reported that HAdV-specific neutralized antibodies mainly target the hexon protein, especially multiple hexon hypervariable regions (41, 42). However, HAdV-specific neutralized antibodies against the fiber knob and penton base have also been described (43). Herein, the recombinant fiber protein of HAdV55 was expressed and purified. MAb 9-8-h2 could bind to HAdV55 virions and fibers, but not to LP12 (**Figure 5A**). MAb 9-8-h2 was also used to identify a conformational epitope common to the HAdV55 fiber. MAb 9-8-h2 could bind HAdV55 and fiber protein under nonreducing conditions (**Figure 5C**), suggesting that mAb 9-8-h2 recognized a conformational epitope in the HAdV55 fiber, which was also present in the HAdV55 trimeric fibers. The trimeric form of the fiber is necessary to induce neutralizing antibodies and to make contact with the receptors, and discontinuous epitopes on the head domain might be involved in fiber-cell interactions (26).

In addition, we used a computational method to analyze the interaction between 9-8-h2 and fiber to locate the binding site, which was supplemented by experimental approaches utilizing targeted mutations. Using computer-generated molecular docking, a model of the interaction between the mAb 9-8-h2 and the HAdV55 fiber was created, and it was suggested that the residues Arg 288, Asn 200, and Asp 157 were important. Our theoretical and experimental approaches were largely convergent, and we discovered that the fiber residues Arg 288, Asn 200 and Asp 157 reacted with CDRH3 and CDRL1 in mAb 9-8-h2. These particular amino acid were essential for this conformational epitope. Next, cryo-EM will be used to investigate the actual crystal structure formed during the binding of the mAb 9-8-h2 to the fiber. The neutralizing antibody mAb 9-8-h2 is a candidate therapeutic antibody; therefore, we will further determine whether it can provide *in vivo* protection against HAdV55. However, currently, we lack an *in vivo* experimental model of HAdV-B infection. Thus, it is important to establish an animal HAdV-B infection model to develop therapeutic antibodies against HAdV-B.

In conclusion, mAb 9-8-h2 had the highest affinity for HAdV55, significantly inhibited the infection of HAdV55 virus at low concentrations *in vitro*, and displayed favorable general physicochemical properties. MAb 9-8-h2 has potential utility for prophylactic or therapeutic purposes against HAdV55 infection. MAb 9-8-h2 should be tested clinically to treat patients, alone or combined with currently available anti-HAdV drugs.

## Data availability statement

The raw data supporting the conclusions of this article will be made available by the authors, without undue reservation.

## Ethics statement

The animal study was reviewed and approved by the Animal Care and Use Committee (Beijing Institute of Biotechnology).

## Author contributions

Conception and design of the experiments: TZ and ZY. Library construction, phage display, cloning, production, purification and characterization of scFv: JG, YJ, KC, and XW. Designed the humanized antibody: JY. Production and purification of recombinant proteins, HAdVs culture, and microneutralization assay: LC. Data analysis and interpretation, figures preparation: JL, RW, YY, and PD. Drafting the manuscript: LC and JL. Revision the manuscript: ZY and TZ. All authors contributed to the article and approved the submitted version.
