# Supplementary material for: A humanized anti-human adenovirus 55 monoclonal antibody with good neutralization ability
Source: Front Immunol. 2023 Mar 16;14:1132822. doi: 10.3389/fimmu.2023.1132822 (PMC10060833; doi:10.3389/fimmu.2023.1132822)
Supplement: Supplementary file 1 [file DataSheet_1.docx]

Supplementary Material

A Humanized Anti-Human Adenovirus 55

Monoclonal Antibody with Good Neutralization Ability

Lei Chen, Jiansheng Lu, Junjie Yue, Rong Wang, Peng Du, Yunzhou Yu, Jiazheng Guo, Xi Wang, Yujia Jiang, Kexuan Cheng, Zhixin Yang*, Tao Zheng*

*** Correspondence:** Tao Zheng: [zhengtao_66@163.com](mailto:zhengtao_66@163.com)

Zhixin Yang, [yy_xiao@126.com](mailto:yy_xiao@126.com)


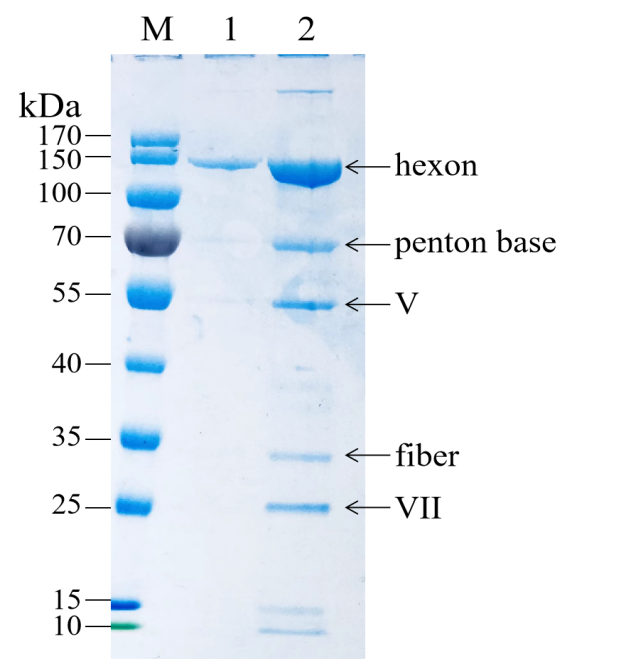


**Supplementary Figure 1. SDS-PAGE of the purified HAdV55.** Purified HAdV55 was mixed with 4×loading buffer, and then heated at 98 °C for 5 min (denatured). M, prestained protein markers; 1, Purified HAdV55 virions; 2, Purified HAdV55 concentrated virions. The corresponding bands were cut and identified by mass spectrometry. The results of the mass spectrometry analysis tentatively determined the attribution and size of the bands of the major structural proteins hexon, fiber and penton in the SDS-PAGE results (data no shown). SDS-PAGE demonstrated that the protein purity of HAdV55 was higher than 95%.


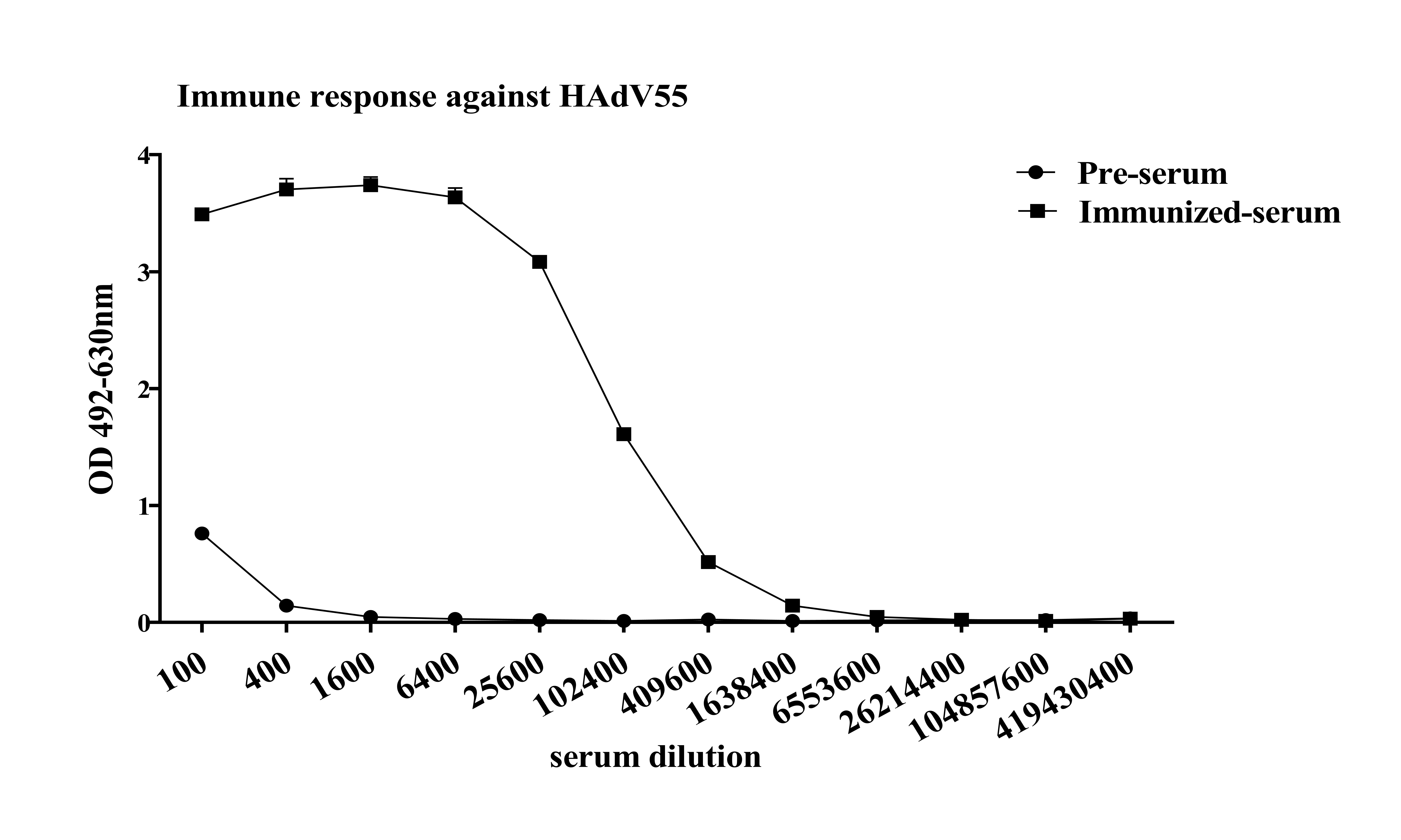


**Supplementary Figure 2.** **Detection of strong and specific serological activities after immunization with HAdV55.** A positive well is typically defined as having a (OD value of positive sera - OD value of blank wells) / (OD value of negative control - OD value of blank wells) ratio > 2.1, with the antibody titer representing the maximum serum dilution in the positive wells.


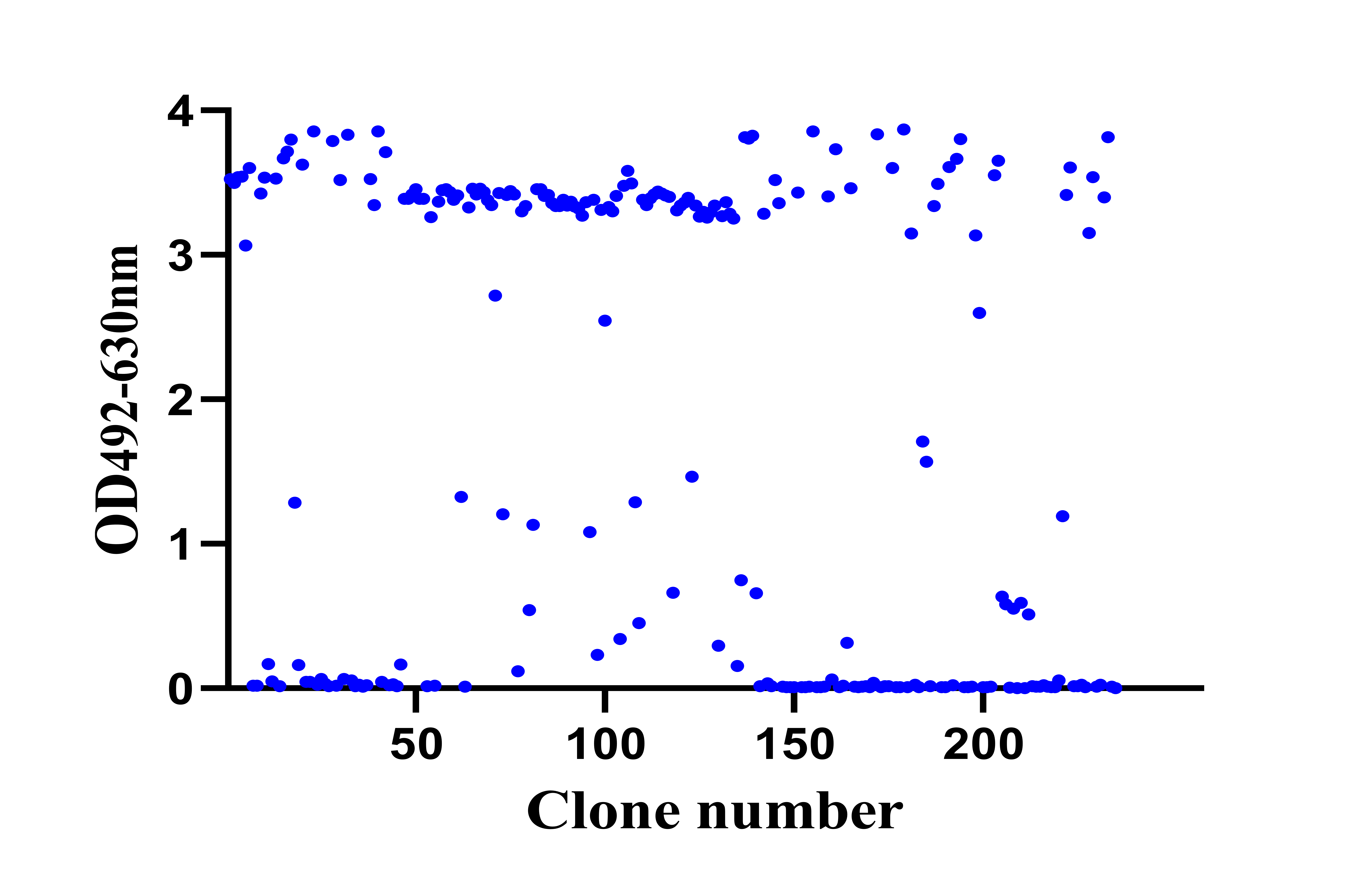


**Supplementary Figure 3.** **Positive clones were identified using PHAGE-ELISA.** A binding ratio > 3 identified a positive clone.


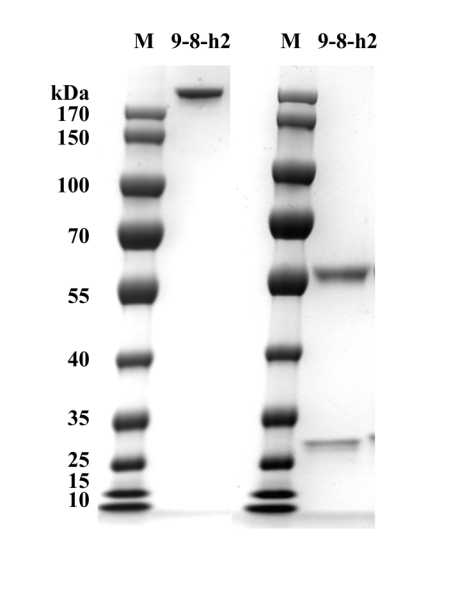


**Supplementary Figure 4.** **SDS-PAGE separation of mAb 9-8-h2 and Coomassie blue staining of affinity purified mAb 9-8-h2 under non-reducing (left) or reducing (right) conditions.**


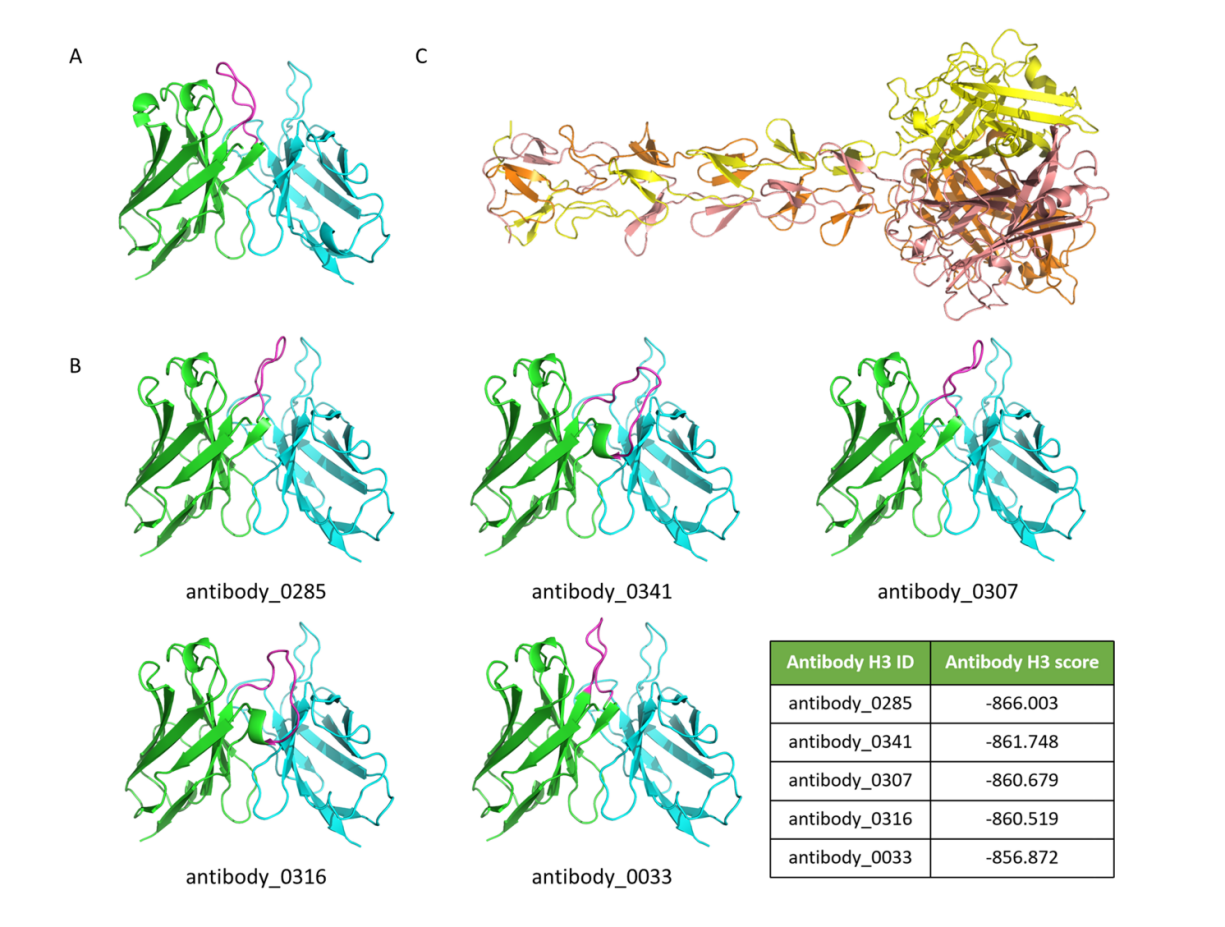


**Supplementary Figure 5.** **Prediction of antibody and antigen 3D structure. (A)**The best predicted model of antibody generated by AlphaFold2. The CDR3 region of heavy chain was marked as magenta. **(B)** Top five antibody structures and its ranking score generated by Antibody_H3 optimization. The CDR3 region was marked as magenta. **(C)** The best predicted model of antigen generated by AlphaFold2.


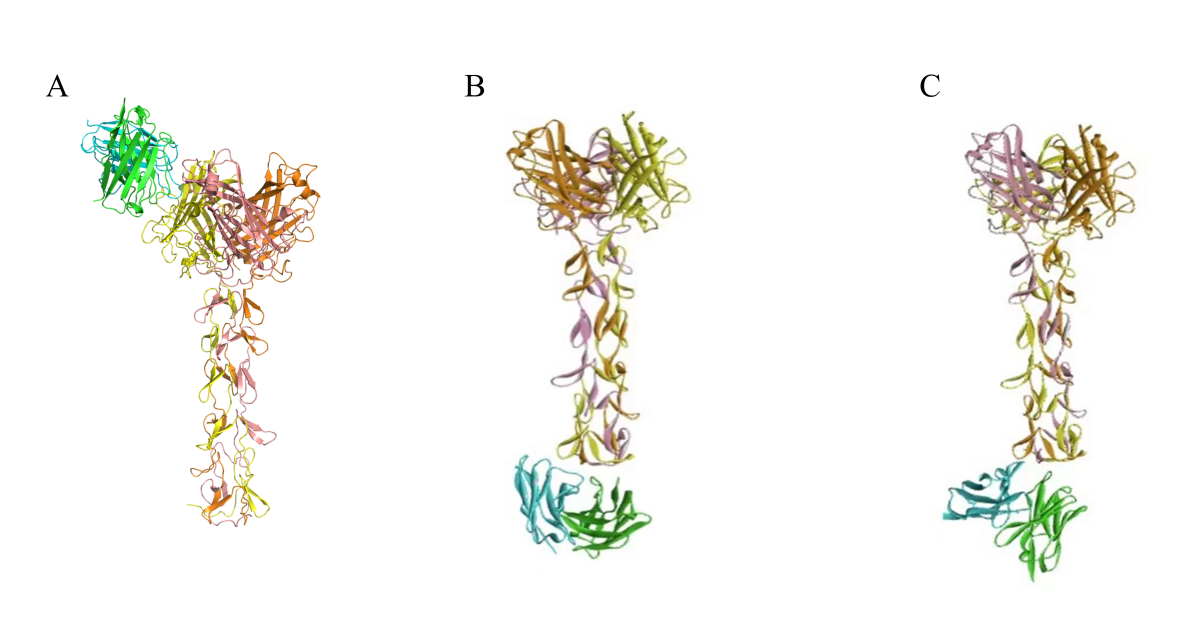


**Supplementary Figure 6.** **The three structures of Antibody_H3 simulation/antigen complex. (A)** The structure of Antibody_0307 / fiber complex. **(B)** The structure of Antibody_0341 / fiber complex. **(C)** The structure of Antibody_0033 / fiber complex.


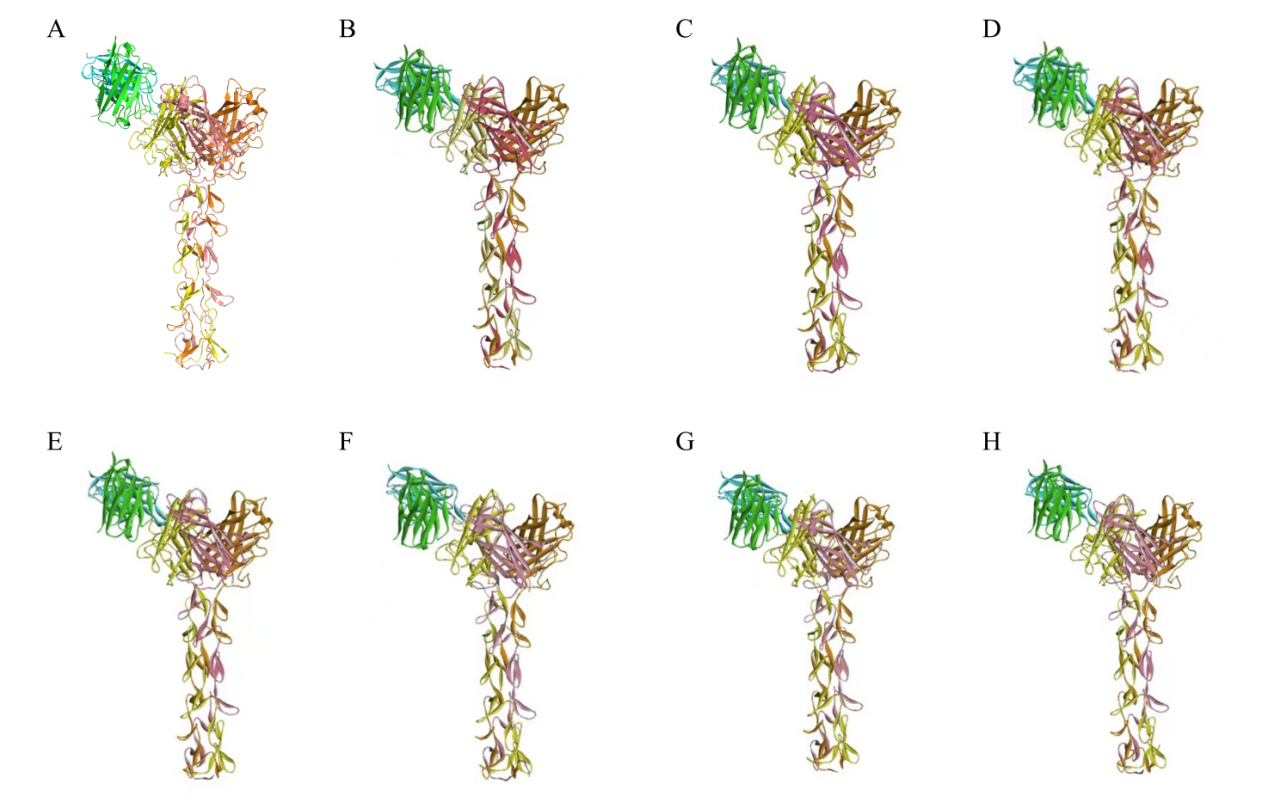


**Supplementary Figure 7.** **The images of the eight similar conformations from the antibody_0307. (A)** The structure of cluster_2_model_1 / fiber complex. **(B)** The structure of cluster_2_model_2 / fiber complex. **(C)** The structure of cluster_2_model_3 / fiber complex. **(D)** The structure of cluster_2_model_4 / fiber complex. **(E)** The structure of cluster_2_model_5 / fiber complex. **(F)** The structure of cluster_2_model_6 / fiber complex. **(G)** The structure of cluster_2_model_7 / fiber complex. **(H)** The structure of cluster_2_model_8 / fiber complex.

**
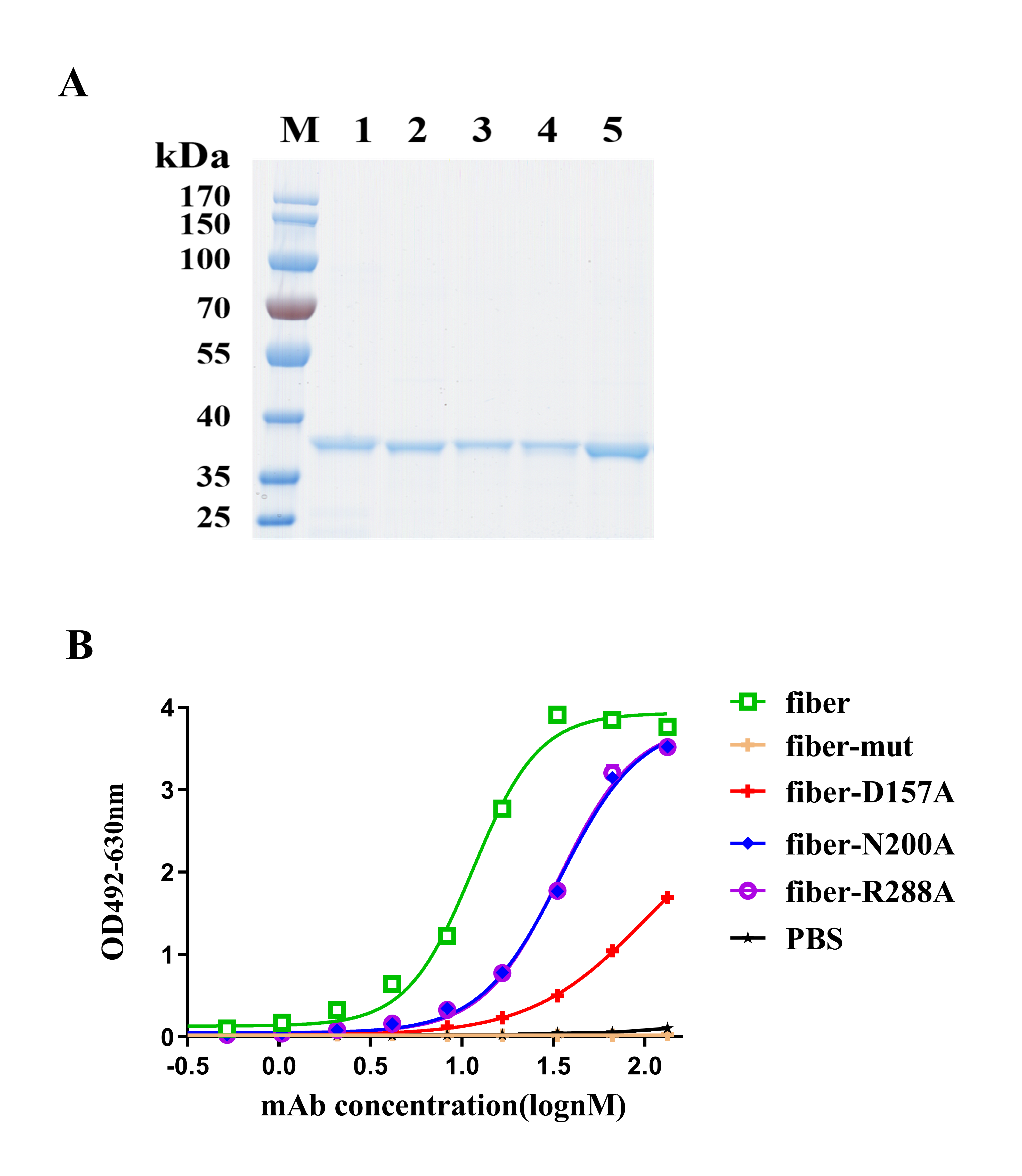
**

**Supplementary Figure 8. Fiber mutants binding to 9-8-h2. (A)** SDS-PAGE analysis of the purified recombinant fiber mutants. M, prestained protein markers; 1, Purified fiber; 2, Purified fiber-D157A; 3, Purified fiber-N200A; 4, Purified fiber-R288A; 5, Purified fiber-mut. **(B)** Fiber mutants binding were determined by ELISA.

**Supplementary Table1.** Neutralizing activities of the HAdV55-specific mAbs evaluated in vitro.

| Antibody  （1332 nM） | Cell Survival Rate (Surviving cell/total cell) | | |
| --- | --- | --- | --- |
|  | HAdV55  （100 TCID50） | HAdV7  （100 TCID50） | HAdV4  （100 TCID50） |
| 9-8 | 4/4 | 0/4 | 0/4 |
| 2-8 | 4/4 | 0/4 | 0/4 |
| 10-4 | 4/4 | 0/4 | 0/4 |
| 12-6 | 0/4 | 0/4 | 0/4 |
| 12-1 | 3/4 | 0/4 | 0/4 |
| 6-8 | 0/4 | 0/4 | 0/4 |
| 7-1 | 0/4 | 0/4 | 0/4 |
| 1-2 | 0/4 | 0/4 | 0/4 |
| 1-5 | 0/4 | 0/4 | 0/4 |
| 8-2 | 2/4 | 0/4 | 0/4 |
| anti-HAdV55 serum | 4/4 | 0/4 | 0/4 |
| anti-EGFR | 0/4 | 0/4 | 0/4 |
| Cell | 4/4 | 4/4 | 4/4 |
| Virus | 0/4 | 0/4 | 0/4 |

**Supplementary Table2.** Results of scanning mutagenesis of FR residues produced by Hu-mAb.

| Chain | Mutations | Residue Number | Original AA | Mutated AA | Humanness Score |
| --- | --- | --- | --- | --- | --- |
| Heavy chain | 1 | 1 | E | Q | 0.125 |
|  | 2 | 12 | L | V | 0.185 |
|  | 3 | 45 | T | A | 0.265 |
|  | 4 | 96 | S | G | 0.335 |
|  | 5 | 101 | M | V | 0.410 |
|  | 6 | 20 | K | R | 0.515 |
|  | 7 | 49 | R | G | 0.635 |
| Light chain | 1 | 9 | S | D | 0.165 |
|  | 2 | 94 | V | L | 0.285 |
|  | 3 | 19 | V | A | 0.410 |
|  | 4 | 15 | V | L | 0.535 |
|  | 5 | 99 | L | V | 0.665 |

**Supplementary Table3.** The Tm&Tagg 266 of humanized mAb 9-8-h2.

| Ab | Tm1 (°C) | | | Tm2 (°C) | | | Tagg (°C) | | |
| --- | --- | --- | --- | --- | --- | --- | --- | --- | --- |
|  | Tm1 | Average | %CV | Tm2 | Average | %CV | Tagg | Average | %CV |
| 9-8-h2 | 67.68 | 67.47 | 0.45 | 76.50 | 76.70 | 0.37 | 65.50 | 65.70 | 0.43 |
|  | 67.25 |  |  | 76.90 |  |  | 65.90 |  |  |

**Supplementary Table4.** The HADDOCK score and confidence scores of the top five structures of Antibody_H3 simulation/antigen complex.

| Antibody-antigen complex | Docking score | Confidence score |
| --- | --- | --- |
| Antibody_0307 / fiber | -260.84 | 0.902 |
| Antibody_0341 / fiber | -160.32 | 0.432 |
| Antibody_0033 / fiber | -140.76 | 0.357 |

**Supplementary Table5.** The docking and confidence scores of the eight similar conformations from the antibody_0307.

| Rank | Docking score | Confidence score |
| --- | --- | --- |
| cluster_2_model_1 | -260.84 | 0.902 |
| cluster_2_model_2 | -249.75 | 0.880 |
| cluster_2_model_3 | -247.98 | 0.877 |
| cluster_2_model_4 | -241.22 | 0.861 |
| cluster_2_model_5 | -239.89 | 0.858 |
| cluster_2_model_6 | -238.32 | 0.854 |
| cluster_2_model_7 | -236.38 | 0.849 |
| cluster_2_model_8 | -235.55 | 0.847 |
